# Metastatic cardiac tumor presenting as atrial fibrillation in a previously healthy woman: A case report

**DOI:** 10.1097/MD.0000000000007649

**Published:** 2017-08-04

**Authors:** Chi-Wen Cheng, Ning-I. Yang, Koon-Kwan Ng, Wen-Jin Cherng

**Affiliations:** a Division of Cardiology, Department of Internal Medicine, Chang Gung Memorial Hospital, Keelung; b Chang Gung University College of Medicine, Taoyuan; c Department of Radiology, Chang Gung Memorial Hospital, Keelung, Taiwan.

**Keywords:** atrial fibrillation, left atrium, metastasis, pulmonary vein

## Abstract

**Rationale:**

Metastatic cardiac tumor (MCT) is rare in clinical practice. MCT presenting initially as atrial fibrillation (AF) is even rarer.

**Patient concerns:**

We report a 47-year-old woman with no previous medical history presented with intermittent palpitation for 3 days.

**Diagnoses:**

The electrocardiography showed AF with rapid ventricular rate. The transthoracic echocardiography showed a 4 × 4 cm mass occupying the left atrium (LA). The contrast enhanced computed tomography (CT) showed a left lower lung mass with invasion to the LA and left upper pulmonary vein (PV). The chest CT guided biopsy revealed poorly differentiated squamous cell carcinoma. Further workup including bone scan showed no significant findings. The diagnosis of lung squamous cell carcinoma with cardiac invasion was made.

**Interventions:**

She went on to received palliative chemotherapy.

**Outcomes:**

She is being followed up regularly at the outpatient department.

**Lessons:**

Tumor invasion of the LA and PV was thought to be the cause of the AF. This condition is rare, but clinically important. Physicians should be alert that MCT could be an important differential diagnosis in patients presenting with unexplained AF.

## 1 Introduction

Though metastatic cardiac tumor (MCT) is rare in clinical practice, the true incidence reported in literature is higher than expected and present in up to 21% of postmortem patients who had died of malignancies.^[1]^ Only about one-tenth of patients who died of tumor disease showed cardiac spread at postmortem examination had presented with cardiac involvement symptoms or findings.^[2]^ Most of the clinical presentations in these patients are dominated by the systemic spread of cancer. The symptoms and signs of cardiac involvement are hence often overlooked. Always being alert of the possibility of MCT is, therefore, very important even if no obvious symptoms or signs are present. Herein, we report a 47-year-old woman with no previous medical history presenting with atrial fibrillation (AF). Lung cancer invading into the left atrium (LA) and pulmonary vein (PV) was detected after a series of investigations.

## 2 Case report

**Figure 1:**
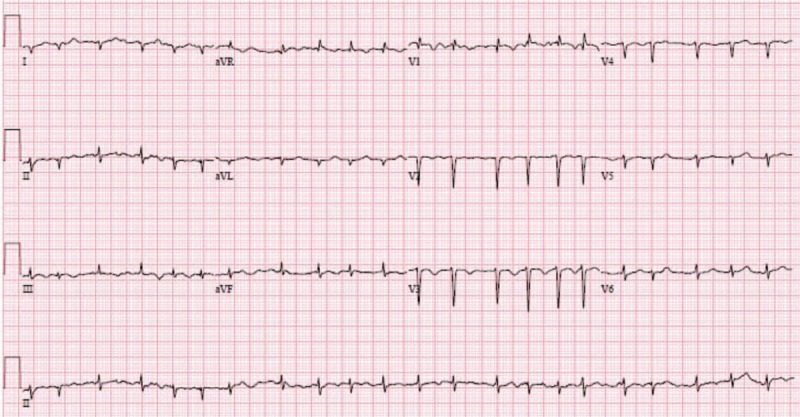
Electrocardiography. Electrocardiography shows atrial fibrillation with rapid ventricular rate, low voltage over limb leads, nonspecific ST-T changes, poor R wave progression, and right axis deviation.

**Figure 2:**
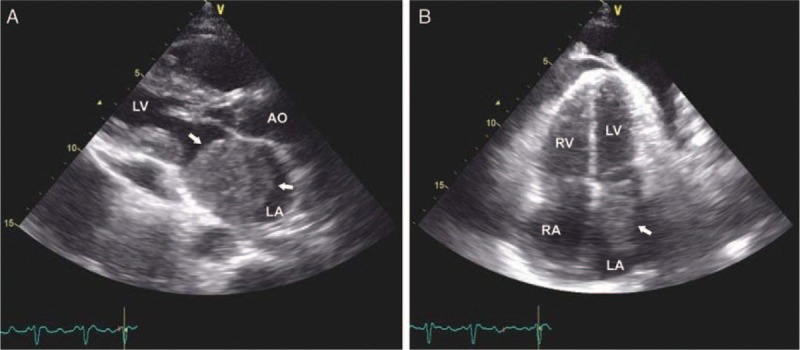
Transthoracic echocardiography. Transthoracic echocardiography in (A) parasternal long-axis and (B) apical 4 chamber views show a mass occupying the left atrium (arrows) and small amount pericardial effusion. AO = aortic root, LA = left atrium, LV = left ventricle, RA = right atrium, RV = right ventricle.

A 47-year-old woman with no previous medical history presented with intermittent palpitation for 3 days. She had none productive cough for 2 weeks and was intermittently treated as having an upper respiratory tract infection at a local medical facility. She was brought to our emergency room due to palpitation and progressive dyspnea. Her heart rate was 132 beats per minute, blood pressure 98/55 mm Hg, respiratory rate 18 breaths per minute. Breath sounds were decreased over the left lower lung area and her heartbeat was irregularly irregular. Other physical examination findings were unremarkable. Her electrocardiogram (EKG) showed AF with rapid ventricular rate (Fig. 1) and chest x-ray showed cardiomegaly with left pleural effusion. Her blood tests including renal, liver, and thyroid function were normal. The transthoracic echocardiography showed a 4 × 4 cm mass occupying the LA (Fig. 2), small amount pericardial effusion, mild tricuspid regurgitation with pressure gradient 43 mm Hg and normal ejection fraction. The contrast-enhanced neck, chest, and abdomen computed tomography (CT) showed a left lower lung mass with invasion to the LA and left superior PV. CT guided biopsy showed poorly differentiated squamous cell carcinoma. Lung squamous cell carcinoma was suspected as the primary tumor. Further workup including bone scan showed no significant findings. Therefore, the diagnosis of lung squamous cell carcinoma with cardiac invasion was made. She went on to received palliative chemotherapy and is being followed up regularly at the outpatient department.

## 3 Discussion

**Figure 3:**
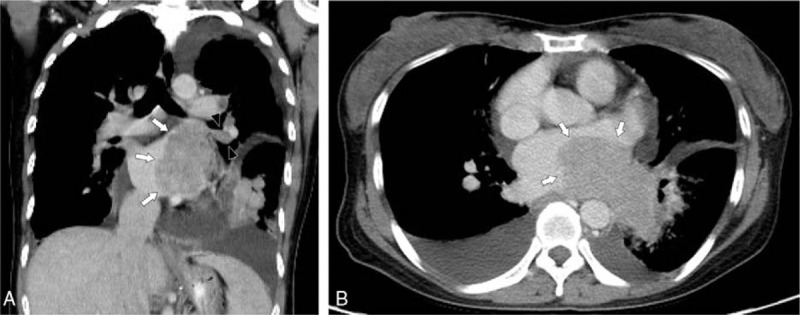
Contrast-enhanced computed tomography. Contrast-enhanced computed tomography in (A) axial and (B) coronal images show tumor invasion of left atrium (arrows) and left superior pulmonary vein (arrow heads).

The MCT reaches the heart via hematogenous or lymphatic spread or by direct or transvenous invasion. Tumors may invade the heart by direct extension but they predominantly attain to the heart by lymphatics.^[2]^ Endocardial metastases are usually the result of the hematogenous spread with intracardiac chambers growth.^[3]^ In our case, the metastatic pathway could be via mediastinal lymph nodes, direct extension to LA (Fig. 3B) and hematogenous spread through left superior PV (Fig. 3A, arrow heads) with intra LA growth (Fig. 2).

The diagnosis of MCT is challenging due to the variety of clinical presentations. It may remain clinically silent and only be diagnosed after death. It also may cause medical emergencies such as cardiac tamponade, heart failure, myocardial infarction and sudden death according to the involved cardiac areas.^[2]^ Echocardiography, CT, and magnetic resonance imaging are feasible diagnostic tools. However, they are limited by their cost and the diagnosis remains late. EKG could be a practical tool for early screening. Cates et al^[4]^ reported that the EKG changes most commonly seen in MCT are T wave inversion, ST segment elevation, new atrial arrhythmia, and low voltage. They concluded that any new EKG changes should raise the suspicion of cardiac metastases in clinically stable patients with cancer and no cardiac symptoms suggestive of ischemia. We have reported a 47-year-old woman with no previous medical history presenting with AF. Following a series of investigations, MCT was detected. From our experience, it is suggested that any new EKG changes should raise the suspicion of cardiac metastases not only in patients with preexisting cancer but also in previously healthy individuals with unexplained AF.

AF is well known to be associated with a variety of medical conditions, such as ageing, hypertension, heart failure, valvular heart diseases, cardiomyopathies, congenital heart defects, coronary artery diseases, thyroid dysfunction, obesity, diabetes mellitus, chronic obstructive pulmonary disease, sleep apnea, and chronic renal disease.^[5]^ However, AF associated with MCT is rarely reported. Goldberger and Ludwig^[6]^ reported a case with a premortem diagnosis of right atrial metastasis from bronchogenic carcinoma and atrial arrhythmias. Yu et al^[7]^ reported a case of known testicular carcinoma presenting as AF and eventually found to have pulmonary metastasis and LA invasion. Shalaby et al^[8]^ reported of a case of recurrent hypopharynx squamous cell carcinoma with invasion into left inferior PV and LA presenting as atrial flutter. What makes our case unique compared with the above cases is AF was the main clinical presentation prior to the diagnosis of malignancy.

Rapidly firing ectopic foci originating in the PV and atrium, together with the autonomic nerves at the PV–LA junction have all been implicated in the genesis of AF.^[9,10]^ In addition, atrial arrhythmias induced by LA mechanical stimulation when food passes through the esophagus or by activation of the autonomous nervous system have also been reported.^[11]^ The possible pathogenesis of AF occurring in this patient could be (1) the enhanced micro-reentry activity when tumor cells infiltrate the LA and PV. (2) Direct local LA mechanical stimulation or activation of the autonomic nerve system. Although it is difficult to be sure how the carcinoma enhances AF activity, it is reasonable to believe when the cancer cells infiltrate normal cardiac cell, the heterogeneous tissue promotes subtract for AF perpetuation. It may well be that alteration in the local autonomic ganglionic plexus and mechanical stretching of LA and PV by tumor could be part of the AF pathogenesis in our case.

In conclusion, AF can be the main presentation of MCT. This condition is rare, but clinically significant. The physician should keep this important differential diagnosis in mind when patients have unexplained AF.

## 4 Ethics

This case report was carried out in accordance with the principles of the Declaration of Helsinki and approved by the Institutional Review Board of Chang Gung Medical Foundation.
